# A new midshelf record in the northern Bay of Biscay (NE Atlantic, CBT-CS11 core): Sedimentological, geochemical and palynological data over the last 7 kyrs

**DOI:** 10.1016/j.dib.2020.105323

**Published:** 2020-02-25

**Authors:** Aurélie Penaud, Axelle Ganne, Pierre-Olivier Coste, Maïwenn Herlédan, Matthieu Durand, Meryem Mojtahid, Jean Nizou, Samuel Toucanne

**Affiliations:** a Univ Brest (UBO), CNRS, UMR 6538 Laboratoire Géosciences Océan (LGO), F-29280, Plouzané, France; b LPG-BIAF UMR-CNRS 6112, Univ Angers, Univ Nantes, CNRS, UFR Sciences, 2 Bd Lavoisier, F-49045, Angers, France; c Ifremer, Géosciences Marines, Centre de Bretagne. ZI Pointe du diable, CS 10070, F-29280, Plouzané, France

**Keywords:** Holocene, NE Atlantic Ocean, Pollen assemblages, Dinoflagellate cyst assemblages, Stable isotopes, Grain-size analysis, XRF

## Abstract

The high-time resolution (∼70 years in average) multi-proxy analysis conducted on the mid-shelf core CBT-CS11 (47°46.429′N; 4°25.308′W; 73 m depth; 3.96 m long; NW France, S Brittany) revealed the complexity of the palaeohydrological and palaeoclimatic signals recorded over the last 7 kyrs in the recently published paper: “Oceanic *versus* continental influences over the last 7 kyrs from a midshelf record in the northern Bay of Biscay (NE Atlantic)” [1]. This study presents the whole CBT-CS11 dataset discussed in [1] including sedimentological (XRF and grain-size (total from [1] and CaCO_3_-free from [2]) analyses), geochemical (oxygen and carbon stable isotopes on two different benthic foraminiferal species: *Ammonia falsobeccarii* from [1] and *Cibicides refulgens* from [2]) analyses) as well as palynological (dinoflagellate cyst and pollen assemblages from [1]) data. The present study also describes the different statistical tests from which ecological groups have been established from palynological indicators in [1].

**Table:** Specifications Table

Subject	Global and Planetary Change
Specific subject area	Land-sea information on the northern Bay of Biscay over the last 7000 years are based on palynomorphs, sedimentology and stable isotopes and related to climate and human forcings.
Type of data	1 Excel File 5 Figures
How data were acquired	- CABTEX cruise (Ifremer; PI: S. Toucanne) with CBT-CSx cores: [3], followed by core opening and XRF analysis (Ifremer GM, Plouzané): J. Nizou and S. Toucanne → [2] for methodology - Sampling for AMS-^14^C datations: A. Ganne and A. Penaud → 25 dates: 23 from ARTEMIS and 2 from Poznán: Table 1 in Ref. [1] - First age model in Ref. [2] improved in Ref. [1] with 4 more radiocarbon dates: A. Penaud and M. Mojtahid → Final age model established with Bchron package in R version 3.5.1 [1] - Palynological treatment of sediments: EPOC palynological laboratory (UMR 5805 CNRS, Bordeaux University) → [1] for methodology - Palynological determinations with Leica microscope DM2500 at X630 magnification at LGO (UMR 6538 CNRS, Brest University): A. Ganne, A. Penaud, M. Herlédan → [1] for methodology - Stable isotopes at the PSO (IUEM, Brest University) on two benthic foraminiferal species: P.O. Coste, M. Durand, M. Mojtahid and A. Penaud → *Ammonia falsobeccarii* (IRMS platform, [1]) and *Cibicides refulgens* (Gas Bench platform, [2]) - Multivariate analysis (DCA and CCA) performed with Past version 1.75b software [4]: A. Penaud →Methodology and data presented in this DiB paper
Data format	Raw (excel file with raw data: final age model, sedimentology including grain-size and XRF analyses, stable isotopes, pollen and dinoflagellate cyst assemblages) Analyzed (multivariate analysis: Fig. 3, Fig. 4, Fig. 5)
Parameters for data collection	Criteria considered for the data collection: high-time resolution (∼70 years in average) multi-proxy analysis on a midshelf core for the NE Atlantic Ocean
Description of data collection	CBT-CS11 core collection: Calypso-Genavir corer was used during the CABTEX cruise (Ifremer) in June 2010 on board the R/V Pourquoi Pas? [3]
Data source location	CBT-CS11 core is archived at the Ifremer-Géosciences Marines, Plouzané, France (PI: S. Toucanne) CBT-CS11 core coordinates and information: 47°46.429′N; 4°25.308′W; 73 m depth; 3.96 m long
Data accessibility	With the article [1]
Related research article	[1] A. Penaud, A. Ganne, F. Eynaud, C. Lambert, P.O. Coste, M. Herlédan, M. Vidal, J. Goslin, P. Stéphan, G. Charria, Y. Pailler, M. Durand, J. Zumaque, M. Mojtahid, 2020. Oceanic *versus* continental influences over the last 7 kyrs from a midshelf record in the northern Bay of Biscay (NE Atlantic). Quaternary Science Reviews 229, 106–135.

**Table:** 

**Value of the Data** • Inedit high resolution (∼70 years in average) multi-proxy analysis for hydrographical changes on the Bay of Biscay shelf together with vegetation changes in north-western France watersheds. • These data can be used for regional/global synthesis regarding paleoceanographic and palaeoclimatic changes in the North Atlantic basin. • Any researcher wishing to compare his data with those of this study will be able to retrieve the original work files.

## 1 Data description

### 1.1 Sedimentological and foraminiferal isotopic data (refer supplementary data online)

All stable isotopic data from [1,2], as well as XRF ([2]) and grain size analyses (total grain-size from [1] and CaCO_3_-free grain-size from [2]) are available online (excel file, supplementary data).

### 1.2 Palynological data (refer supplementary data online)

All palynological (pollen and dinoflagellate cyst) data from [1] are available online (raw counts in the excel file, supplementary data), so as to be easily extracted and plotted in future studies in percentages/concentrations.

### 1.3 Canonical Correspondence Analysis (CCA)

**Fig. 1:**
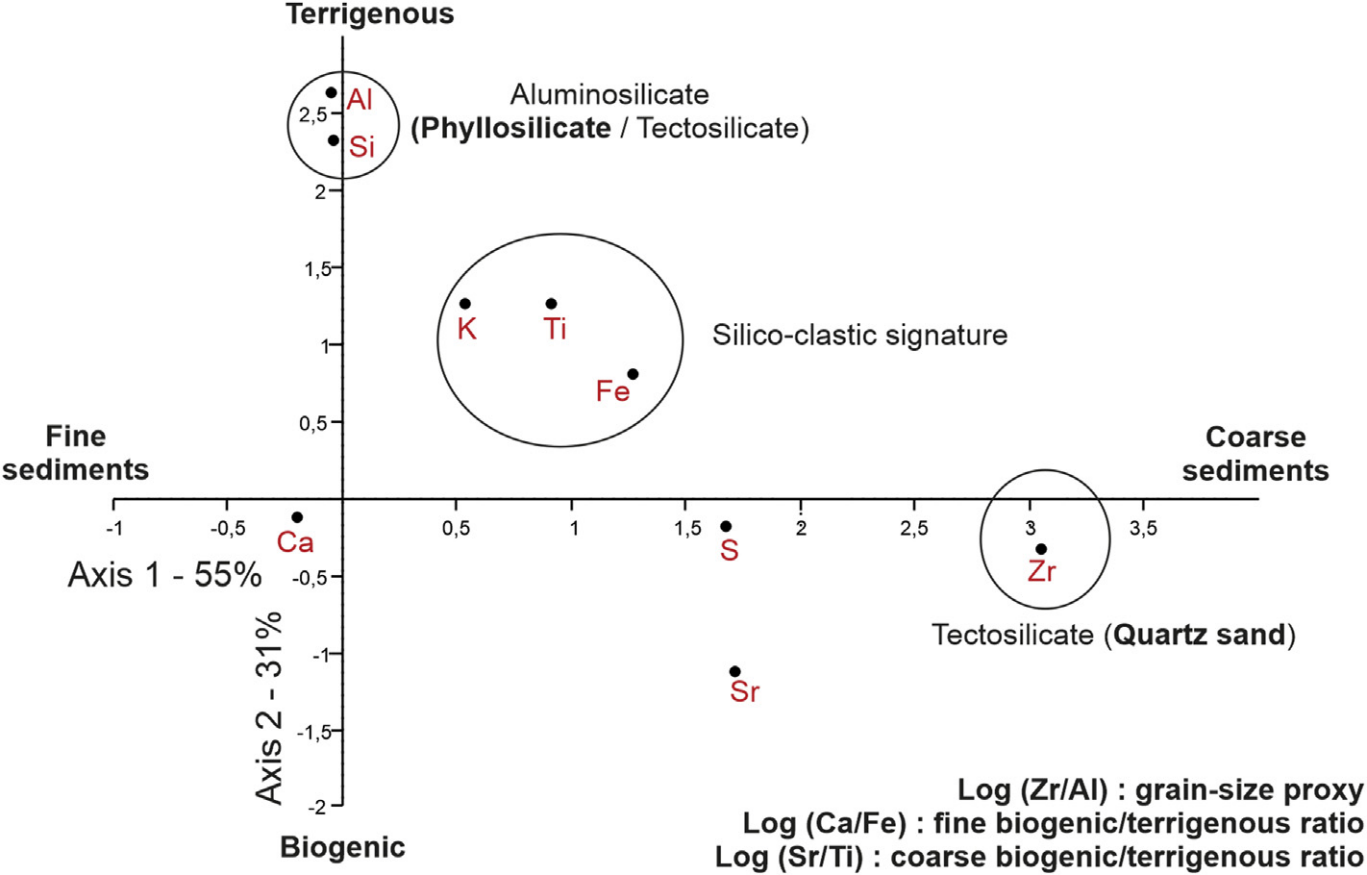
Canonical Correspondence Analysis (CCA) performed on XRF data for CBT-CS11 core.

CCA performed on XRF measurements show chemical elements co-occurrences regarding their environmental signature so as to select main useful data and ratios in [1,2]. On the studied area (silico-clastic margin), K–Ti–Fe elements are related to a detrital terrigenous signature, while Ca–Sr elements are associated to a marine productivity signal [2]. This well-established source of XRF variation is reflected in our data by the second axis on the CCA (Fig. 1). The first axis refers to grain-size properties since Zr is mainly carried by zircon minerals in quartz-rich sands while Al–Si elements are the main elementary components of phyllosilicates and thus potentially of the clayey fraction.

**Fig. 2:**
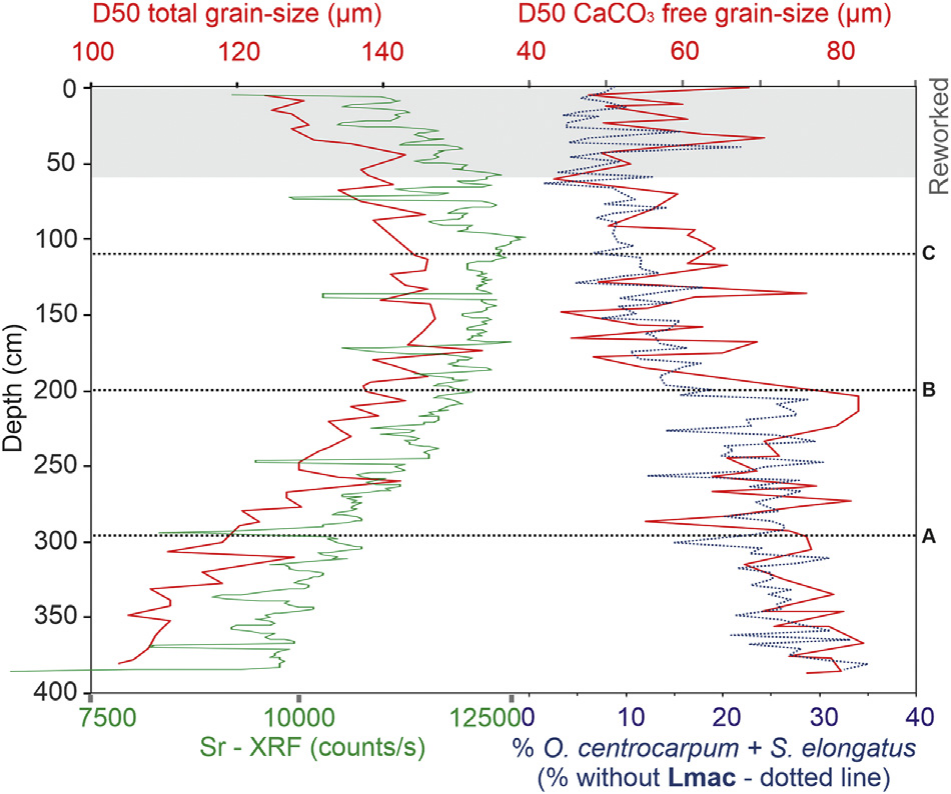
CBT-CS11 core data presented in depth. D50 of total grain-size analyses in parallel with Sr-XRF data, and D50 of CaCO_3_-free grain-size analyses in parallel with sum of subpolar North Atlantic dinoflagellate cyst percentages (*Operculodinium centrocarpum* and *Spiniferites elongatus*) calculated on a dinocyst sum that excudes *Lingulodinium machaerophorum* (Lmac) counts.

We have plotted the D50 from the total grain-size measurements with Sr-XRF counts (Fig. 2). Both are well correlated, reinforcing the idea that Sr can be used as a coarse biogenic tracer.

The Zr/Al ratio discussed in [1] allows deciphering generally coarser (Zr) *versus* finer (Al) sediment composition, while Ca/Fe and Sr/Ti ratios discussed in [2] allow discussing biogenic (Ca and Sr) *versus* terrigenous (Fe and Ti) fractions, with the biogenic component characterized by finer (Ca) or coarser (Sr) biogenic clasts.

Subpolar North Atlantic dinocyst (*Operculodinium centrocarpum* and *Spiniferites elongatus*) percentages [1] show similar pattern with the terrigenous D50 CaCO_3_-free grain-size data from [2]. This dinocyst binome is addressed in [1] for tracing oceanic influence in the mid-shelf studied site.

## 2 Detrended Correspondence Analysis (DCA)

DCA were applied to dinocyst and pollen communities expressed in absolute concentrations in order to capture main factors (environmental variables including grain-size, XRF, and stable isotopic data) that typify both continental and marine studied communities [1].

### 2.1 DCA on dinocyst concentrations (Fig. 3)

- North Atlantic subpolar gyre grouping (*O. centrocarpum* and *S. elongatus*) is explained by grain-size proxies (D50, Zr/Al, Fe/Ca).
- Coastal heterotrophic taxa (*Echinidinium* spp. and *Selenopemphix quanta-Protoperidinium nudum*) are explained by heavier δ^18^O *Cibicides refulgens* [2] that may be related to colder temperatures as also underlined by the proximity of cysts of *Pentapharsodinium dalei* in the biplot.
- The estuarine taxon *Lingulodinium machaerophorum* is individualized from the rest of the dinocyst assemblage. Epiphytic foraminifera [2] explain a large part of this signature, consistently with the alluvial forest signature (i.e., riparian trees, *Alnus* mainly) that may explain the proportion of these foraminiferal taxa first fixed on plants and then transported by fluvial currents.

**Fig. 3:**
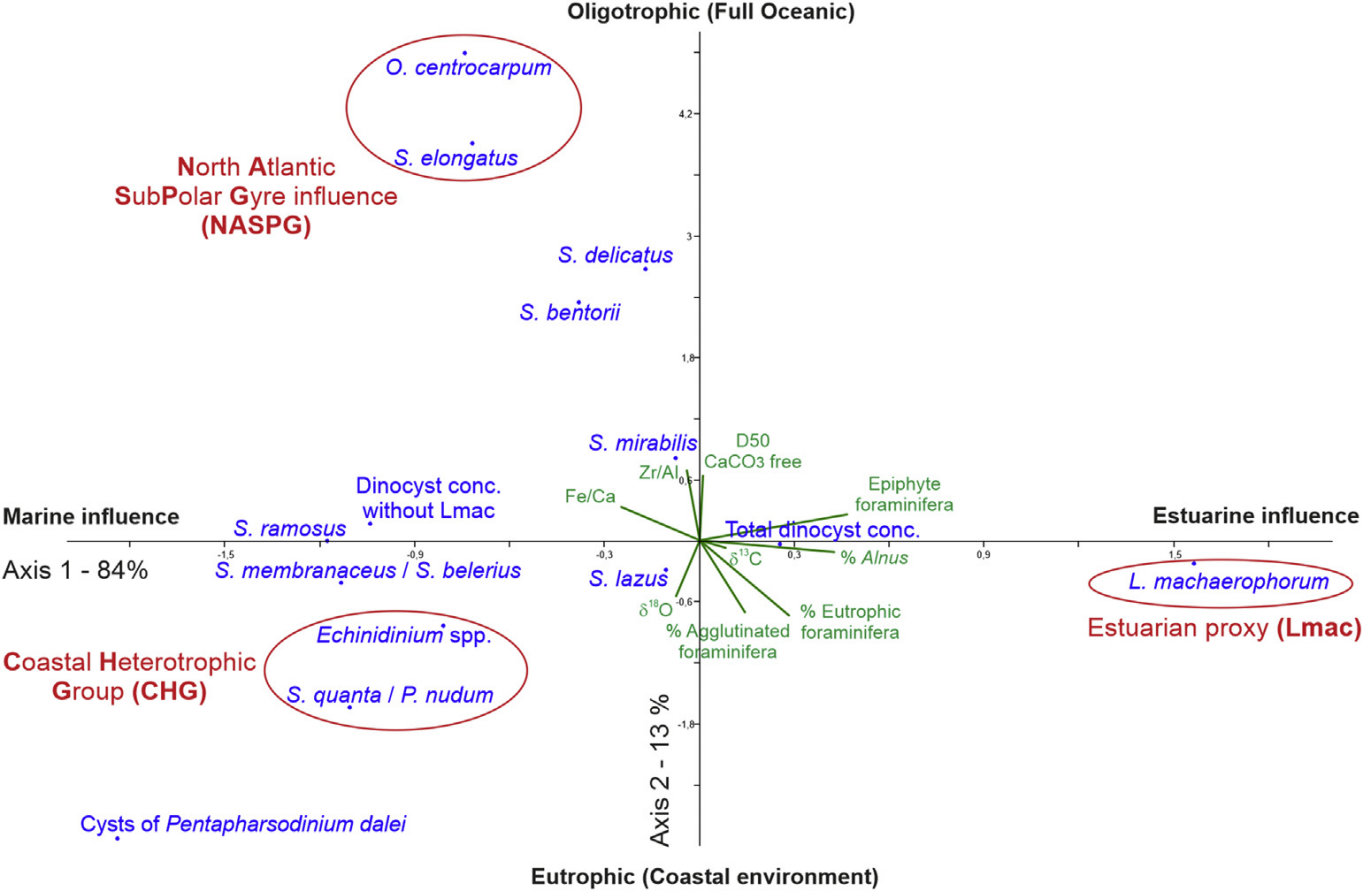
Detrended Correspondence Analysis (DCA) applied to dinocyst absolute concentrations with projected environmental variables including XRF ratios (Fe/Ca, Zr/Al), stable isotopic data on *Ammonia falsobeccarii* (oxygen and carbon), CaCO_3_-free grain-size analyses (D50), benthic foraminiferal percentages (ecological groups from [2]: agglutinated, epiphyte, and eutrophic foraminifera), as well as *Alnus* pollen percentages.

### 2.2 DCA on pollen concentrations (Fig. 4)

- The mixed oak forest (*Quercus* and *Corylus*) grouping covaries mainly with oceanic sedimentological tracers (Zr/Al and D50), suggesting a distal signature (i.e., pole consisting in “marine-influenced” pollinic tracers).
- The alluvial forest (i.e., riparian trees, *Alnus* mainly) signature is explained by enhanced freshwater microalgae (*Halodinium* spp., *Pediastrum* spp., *Botryococcus* spp.) and *L. machaerophorum* percentages.
- Anthropic pollen signature (Poaceae, cultures and ruderal plants with prevailing *Plantago lanceolata*) are explained by higher dinocyst (*L. machaerophorum* excluded) concentrations.

**Fig. 4:**
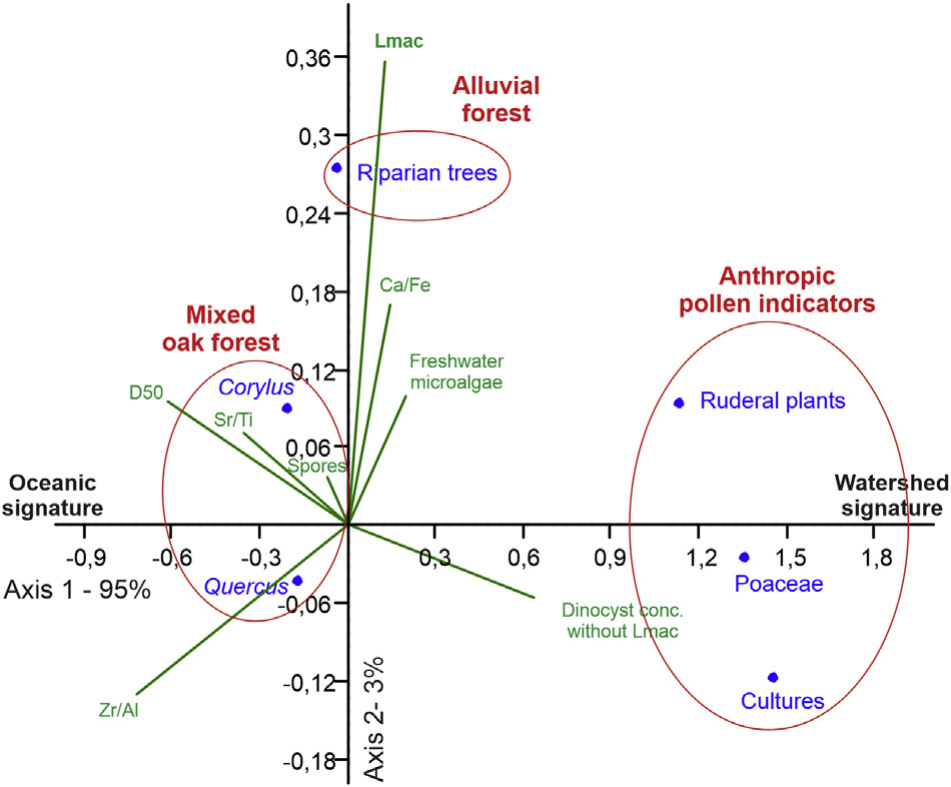
Detrended Correspondence Analysis (DCA) applied to pollen absolute concentrations with projected environmental variables including XRF ratios (Ca/Fe, Sr/Ti, Zr/Al), CaCO_3_-free grain-size analyses (D50), concentrations of: spores, freshwater microalgae, total dinocyst concentrations without *Lingulodinium machaerophorum* (Lmac).

### 2.3 DCA synthesis (Fig. 5)

**Fig. 5:**
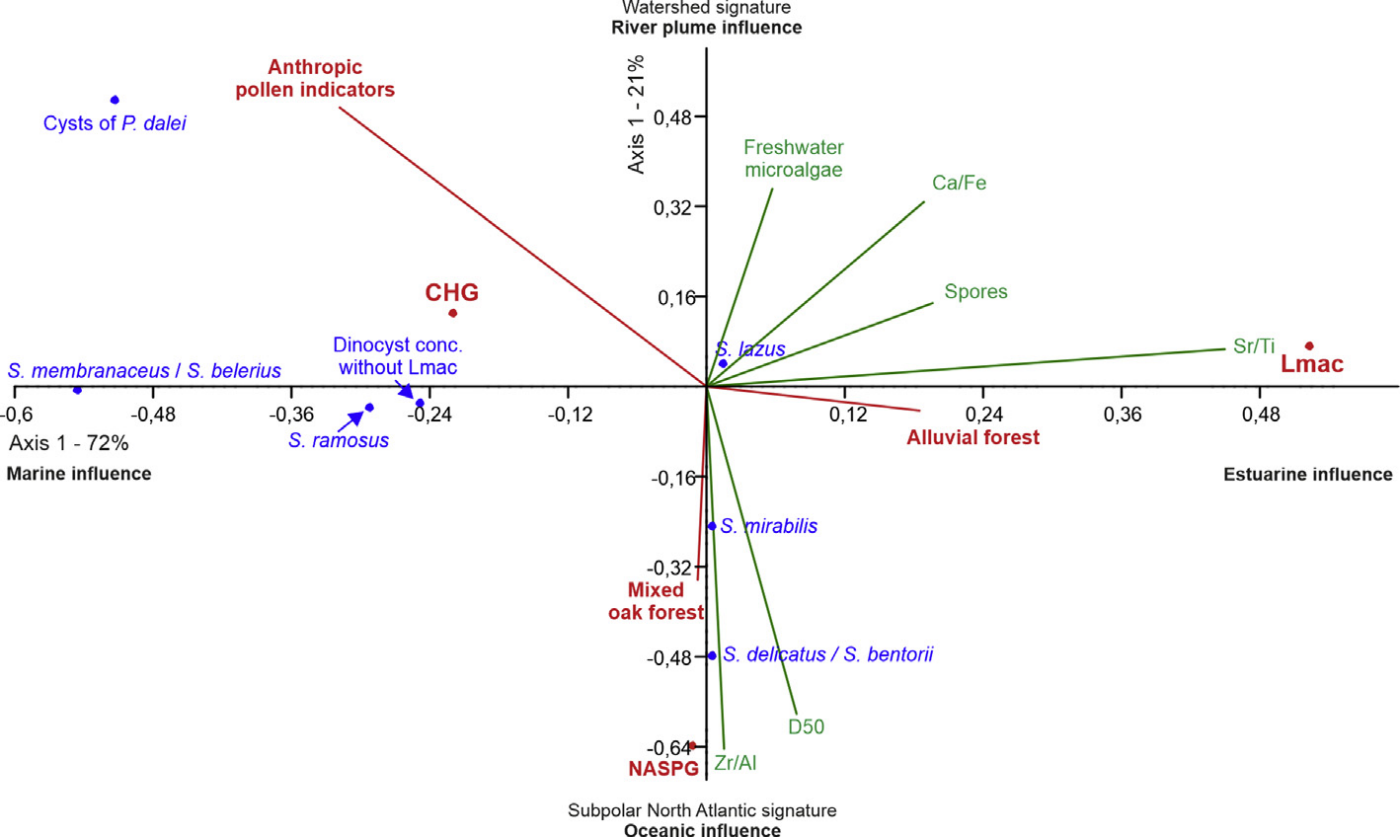
Detrended Correspondence Analysis (DCA) applied to dinocyst absolute concentrations (CHG for Coastal Heterotrophic Group, NASPG for North Atlantic SubPolar Gyre group, Lmac for *Lingulodinium machaerophorum*, cf. Fig. 3) with projected environmental variables including XRF ratios (Ca/Fe, Sr/Ti, Zr/Al), CaCO_3_-free grain-size analyses (D50), as well as concentrations of continental palynomorphs : spores, freshwater microalgae, pollen groups (anthropic pollen indicators, mixed oak forest, alluvial forest, cf. Fig. 4).

Two natural modes (fluvial *versus* oceanic influences) can be distinguished while the third mode characterizes the strongest human impacts on the environment. Fluvial/proximal signature, with finer (Ca) or coarser (Sr) biogenic sediments, characterized by fine detritic sediments, is characteristic of the “*L. machaerophorum* - alluvial forest” pole (top right of Fig. 5). Marine/distal signature is characterized by coarse detritic sediments and is expressed by the “oceanic cysts – mixed oak forest” pole (bottom right of Fig. 5). The anthropic signature (top left of Fig. 5) is individualized by pollen indices of anthropisation combined with heterotrophic cysts and high total cyst (*L. machaerophorum* excluded) concentrations.

## 3 Experimental design, materials, and methods

### 3.1 Sedimentological data [2]

Grain-size and XRF data are fully described in [2] and briefly described in Table 2 of [1].

### 3.2 Stable isotopes [1,2]

*Ammonia falsobeccarii* [1] (101 analyses) and *Cibicides refulgens* [2] (113 analyses) were hand-picked in the 150–250 μm fraction. Prior to isotopic analyses, foraminifera were cleaned in a methanol ultrasonic bath for a few seconds, and then roasted under vacuum at 380 °C for 45 min to remove organic matter. The δ^13^C and δ^18^O (‰VPDB) were measured at the PSO (IUEM, Brest) using the IRMS platform (MAT253 mass spectrometer coupled with a KIEL IV preparation line for benthic species) for [1] and the Gas Bench platform for [2]. The external reproducibility (1σ) on repeated measurements of NBS19 international standard is ±0.04‰ and 0.09‰ for δ^13^C and δ^18^O, respectively.

### 3.3 Palynological analyses [1]

Palynological treatments (EPOC laboratory, Bordeaux University) were conducted on the <150 μm fraction under the following protocol [5]: 3–5 cm^3^ of dry sediments were first treated at room temperature by hydrochloric acids (cold HCl: 10-25-50% until the reaction is over and the effervescence disappears) and hydrofluoric acids (cold HF: 45% for 4 hours followed by 70% for 30h) to remove carbonates and silicates, respectively, then sieved through 10 μm nylon mesh screens.

Palynomorph (i.e. organic microfossils fossilized and observed on palynological slides) determinations were made using a Leica microscope DM2500 at X630 magnification. A minimum of 300 dinocysts and 200 pollen grains were counted per level. In case of over-representation of a taxon (here *Lingulodinium machaerophorum* for dinocyst assemblages), counts were prolonged until at least 100 other specimens were found.

### 3.4 Multivariate analyses

Multivariate analyses were performed on CBT-CS11 core: Canonical Correspondence Analysis (CCA) for XRF data and Detrended Correspondence Analysis (DCA) for palynological data (dinoflagellate cyst and pollen total and specific absolute concentrations, in parallel with environmental variables including grain-size, XRF, and stable isotopic data) thanks to the “Past version 1.75b” software [4].

## References

[1] Penaud A., Ganne A., Eynaud F., Lambert C., Coste P.O., Herlédan M., Vidal M., Goslin J., Stéphan P., Charria G., Pailler Y., Durand M., Zumaque J., Mojtahid M. (2020). Oceanic *versus* continental influences over the last 7 kyrs from a midshelf record in the northern Bay of Biscay (NE Atlantic). Quat. Sci. Rev..

[2] Mojtahid M., Durand M., Coste P.O., Toucanne S., Howa H., Nizou J., Eynaud F., Penaud A. (2019). Millennial-scale Holocene hydrological changes in the northeast Atlantic: new insights from ‘La Grande Vasière’ mid-shelf mud belt. Holocene.

[3] Dussud L. (2010). CABTEX cruise, RV Pourquoi pas ?. Sismer.

[4] Hammer Ø., Harper D.A.T., Ryan P.D. (2001). Past: paleontological statistics software package for education and data analysis. Palaeontol. Electron..

[5] de Vernal A., Henry M., Bilodeau G. (1999). Technique de préparation et d’analyse en micropaléontologie.

